# Prevalence of p.V37I Variant of GJB2 in Mild or Moderate Hearing Loss in a Pediatric Population and the Interpretation of Its Pathogenicity

**DOI:** 10.1371/journal.pone.0061592

**Published:** 2013-04-25

**Authors:** So Young Kim, Gibeom Park, Kyu-Hee Han, Ahreum Kim, Ja-Won Koo, Sun O. Chang, Seung Ha Oh, Woong-Yang Park, Byung Yoon Choi

**Affiliations:** 1 Department of Otorhinolaryngology-Head and Neck Surgery, Seoul National University Hospital, Seoul National University College of Medicine, Seoul, Korea; 2 Department of Biomedical Sciences, Seoul National University College of Medicine, Seoul, Korea; 3 Department of Otorhinolaryngology-Head and Neck Surgery, Seoul National University Bundang Hospital, Seoul National University College of Medicine, Seongnam, Korea; 4 Sensory Organ Research Institute, Seoul National University Medical Research Center, Seoul, Korea; 5 Department of Molecular Cell Biology, Sungkyunkwan University School of Medicine, Suwon, Korea; 6 Translational Genomics Laboratory, Samsung Genome Institute, Samsung Medical Center, Seoul, Korea; Centers for Disease Control and Prevention, United States of America

## Abstract

A p.V37I variant of GJB2 has been reported from subjects with moderate or slight hearing loss especially in East Asian populations. This study aimed to estimate the prevalence of the p.V37I variant among such subjects and prove, epidemiologically, its pathogenic potential to cause mild hearing loss. A total of 380 subjects from 201 families with hearing loss were enrolled. From them, 103 families were selected who had autosomal recessive inheritance or sporadic occurrence of hearing loss and who were younger than 15 years old. *GJB2* sequencing was carried out for the probands of all 103 families. The prevalence of the p.V37I variant was compared between the subtle, mild or moderate hearing loss (group I) and the severe or profound hearing loss (group II) groups. Where possible, a targeted next generation sequencing of 82 deafness genes was performed from the p.V37I carrier to exclude the existence of other pathogenic genes. Five (4.8%) of 103 probands were found to carry p.V37I. The carrier frequency of p.V37I among group I (18.2%) was significantly higher than that of group II (1.2%) or the reported Korean normal hearing control group (1.0%). Detection of the p.V37I variant of GJB2 in 18.2% of Koreans with mild hearing loss strongly suggests its contribution to the pathogenesis of milder hearing loss, which might justify sequencing of *GJB2* from these subjects in the Korean population.

## Introduction

Despite significant heterogeneity of genetic deafness, *GJB2* (encoding Gap junction beta-2 protein, also known as connexin 26) causes more than 50% of autosomal recessive nonsyndromic deafness in many populations [1], [2]. More than 100 different pathogenic mutations and 24 polymorphisms have been identified within the *GJB2* gene (http://davinci.crg.es/deafness/) [3]. The mutational spectra often vary among different ethnic groups. For example, in Caucasians, the 35delG mutation accounts for up to 85% of the *GJB2* mutant alleles [2], [4], [5]. By contrast, the 235delC mutation is prevalent in Koreans [6], Japanese [7], Chinese [8] and Taiwanese [9].

The p.V37I is another *GJB2* variant that is frequently found in East Asian populations [10]–[13]. The variant resides in transmembrane M1 domain in beta connexins [14]. The p.V37I variant has been frequently observed to be associated with mild to moderate hearing loss [15], [16].

The p.V37I variant was originally described as an innocent polymorphism because it was identified in unaffected heterozygous control subjects [14]. However, with the increasing number of identified hearing impaired subjects carrying the homozygous p.V37I or the heterozygous p.V37I in a *trans* configuration with other pathogenic mutation of *GJB2*, the possibility of a pathogenic role of the variant has been raised [17]–[19]. Recent functional studies have produced somewhat contradictory results regarding the pathogenicity of the p.V37I variant. Functional studies of electrical conductance between paired Xenopus oocytes demonstrated that the p.V37I mutant protein is non-functional, implying that the variant is a pathogenic mutation [20]. In contrast with the null function of the variant proposed by Bruzzone et al. (2003), other recent studies demonstrated only a partially reduced function of the p.V37I mutant protein [21], [22]. These studies showed that the penetrance of the p.V37I variant was only about 1/10 relative to known pathogenic *GJB2* mutations [22].

Recently, an epidemiological study of a Chinese population by Li et al. (2012) demonstrated that the p.V37I variant, either in compound heterozygosity with other truncating mutations of *GJB2* or in homozygosity, may cause subclinical subtle hearing loss at birth, and this hearing loss may progress and become apparent at later age. In that paper, they showed that the genotype is present in a substantial portion (20%) of post natal permanent childhood hearing impairment (PCHI) [23]. Rarer detection of p.V37I variant allele in severe to profound hearing loss than what was predicted from the prevalence of this allele in normal hearing controls in the Korean population [6], [12], [13] strongly suggests that p.V37I variant contributes to milder hearing loss.

To the best of our knowledge, there has been no study estimating the prevalence of the p.V37I variant according to the degree of hearing loss. Therefore, we conducted a genetic test to estimate genetic loads of the p.V37I variants according to the degree of sensorineural hearing loss.

## Methods

### Ethical Considerations

The Institutional Review Boards (IRBs) at the Seoul National University Bundang Hospital (SNUBH) (IRB-B-1007-105-402) and the Seoul National University Hospital (SNUH) (IRBY-H-0905-041-281) approved the study. We obtained a written informed consent from all the participants in this study. In case of children participants, the written informed consent was obtained from the parents or guardians on behalf of them.

### Study Participants

Clinical phenotype evaluations included medical and developmental history interviews, physical examinations, pure tone audiometry and imaging studies (Temporal bone CT and/or MR images), whenever possible. An initial cohort of 380 subjects from 201 families with varying degrees of hearing loss were collected at the otolaryngology clinics of SNUH and SNUBH from September 2010 through though March 2012. The 380 subjects were subjected to preselection based upon the presence of autosomal recessive inheritance or sporadic occurrence of hearing loss. Ten milliliters of whole blood was obtained from all probands in the 201 families and, if possible, their siblings and parents for the segregation study. Families segregating hearing loss in an autosomal dominant, a maternally transmitted or X-linked manner were excluded. To exclude any bias resulting from presbycusis, subjects were excluded if they were older than 15 years of age at their initial visit. Individuals with syndromic, unilateral hearing loss, or with inner ear anomalies, such as enlarged vestibular aqueduct or incomplete partition type III, were also excluded from this study. This preselection resulted in the number of study families being reduced to 103.

### Clinical Evaluation

The following demographic data were collected for each patient: sex, birth date, date of initial otolaryngological consultation, and major comorbidities or syndromic features. The ethnicity of all subjects was Korean. Where available, the temporal bone CT or magnetic resonance imaging findings were also taken. For example, anomalies in the seventh nerve, cochlear anomaly or enlarged vestibular aqueduct syndromes were all recorded.

### Audiometric Evaluation

**Figure 1:**
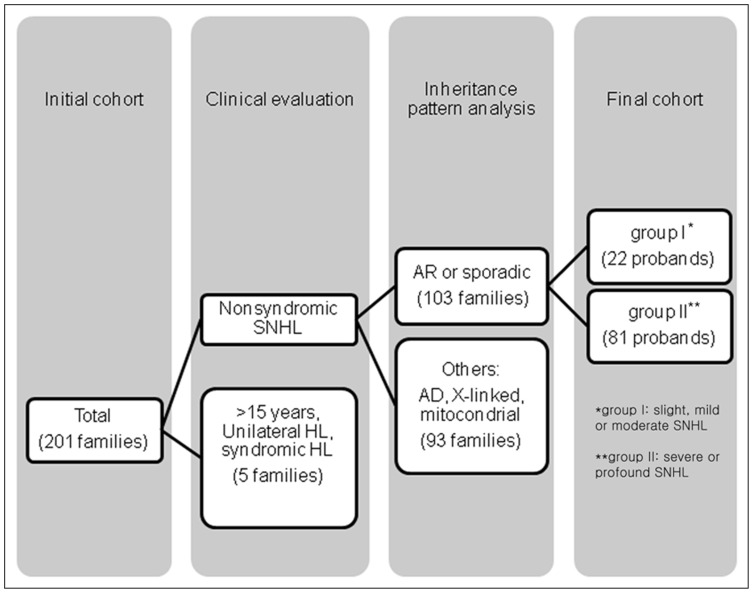
Schematic flow chart of this study. An initial cohort of 201 families underwent genetic studies. Among them, subjects with nonsyndromic SNHL (sensorineural hearing loss) and autosomal recessive (AR) or sporadic inheritance patterns were selected. Based on the degree of hearing loss, final cohorts were classified into two groups: slight, mild or moderate SNHL (group I); severe or profound SNHL (group II).

All subjects underwent audiological evaluation. Hearing levels were determined by pure-tone audiometry. We performed serial audiograms regularly to detect any noticeable aggravation of hearing after their participation in this study and to provide timely auditory rehabilitation. In addition, if necessary, we traced back the previous hearing status by obtaining the clinical information from the private practitioner who referred patients to our center. Initial audiograms conducted in our center were reviewed, and the pure-tone average (PTA) was calculated for each ear. The pure-tone thresholds at 0.5, 1, 2, and 4 kHz were recorded, and the pure-tone average across these frequencies was calculated and reported as decibels of hearing loss. In six cases where pure-tone audiometry was not performed because of the subjects’ young age, auditory brainstem response threshold (ABRT) evaluations and/or auditory Steady-State Response(ASSR) were recorded, and mean thresholds at frequencies in the 0.5-to 4-kHz range were averaged to approximate the PTA. The hearing level of the better ear calculated by four-tone average (0.5, 1, 2, and 4 kHz) was labeled as subtle (16–25 dB), mild (26–40 dB), moderate (41–70 dB), severe (71–95 dB), or profound (>95 dB). This cohort was then screened by visual examination of audiometric data and classified into two groups: Subtle, mild or moderate sensorineural hearing loss (SNHL) (group I) versus severe or profound SNHL (group II) (Figure 1).

### PCR and Sanger Sequencing of *GJB2*

As a first step toward molecular genetic diagnosis of the 103 families, we performed bidirectional nucleotide sequence analysis of the two exons of *GJB2* (GenBank Accession NM_004004.5). The two exons and flanking sequences of *GJB2* were PCR-amplified and sequenced as previously described [12]. Mutation nomenclature and numbering were based upon NM_004004.5, a cDNA for *GJB2*. The names of all variants were checked using Mutalyzer (http://www.LOVD.nl/mutalyzer/). Novel splice site variants and synonymous changes were considered non-pathogenic when they were detected in the normal Korean population or BDGP (http://www.fruitfly.org/seq_tools/splice.html) and ESEfinder (http://rulai.cshl.edu/cgi-bin/tools/ESE3/esefinder.cgi?process=home) software did not predict a change that creates or eliminates a splicing acceptor or donor site. We have submitted our novel variants of *GJB2* to the LSDB database (https://research.cchmc.org/LOVD2/variants.php?select_db=GJB2&action=view&view=0000808).

### Targeted Capture of Exons and Flanking Sequences of 82 Deafness Genes and Massively Parallel Sequencing of DNA Libraries

For two subjects (SH42-94 and SB51-95) carrying the p.V37I variant of *GJB2* as one of two mutant alleles, they were further evaluated by targeted capture of exons and flanking sequences of 82 deafness genes, followed by massively parallel sequencing of DNA libraries, as previously described [24]. We have deposited our whole sequencing data in our private SNUH-SNUBH sequencing database and have submitted the novel variants of *TRIBOP*, *TMC1* and *DSPP* that we detected by next generation sequencing to the LSDB databases (http://grenada.lumc.nl/LOVD2/mendelian_genes/variants.php?select_db=TRIOBP&action=view&view=0059473,https://research.cchmc.org/LOVD2/variants.php?select_db=TMC1&action=view&view=0000809,http://grenada.lumc.nl/LOVD2/mendelian_genes/variants.php?select_db=DSPP&action=view&view=0059474).

### Breakpoint PCR for Detection of known Deletions Involving GJB6

To exclude the possibility of large genomic deletions that may be in a *trans* configuration with p.V37I, we conducted a multiplex breakpoint PCR assay for the single heterozygous carriers of the p.V37I allele designed to detect known reported large genomic deletions (del(GJB6-D13S1830) and del(GJB6-D13S1854)) [25], [26]. The sequences of the primers and PCR conditions were as previously reported [25]. Water was used as a negative control, and two single heterozygous carriers of the p.V37I, SB80-141 in group I, and SHJ1 in group II were examined.

### Statistical Analysis

The SPSS15 statistical package was used for the analysis (SPSS Inc., Chicago, IL, USA). Fisher’s exact test and logistic regression analysis were used to compare the prevalence of the p.V37I variant among different subject groups. For control groups, we assembled a composite cohort of 3418 ethnically matched Korean normal hearing subjects from three independent studies (Han et al. 2008; Kim et al. 2011; Park et al. 2000).

Odds ratios and confidence intervals were calculated by logistic regression analysis; p-values ≤0.05 were considered statistically significant.

## Results

One hundred and three families satisfied our inclusion criteria by showing either autosomal recessive inheritance pattern or sporadic occurrence of the hearing loss (Figure.1). Twenty-two of the 103 families showed moderate or slight hearing loss (group I), while the other 81 families manifested severe hearing loss (group II) (Figure.1). Five (4.8%) of 103 probands carried the p.V37I variant of *GJB2*. The variant was detected in a *trans* configuration with a known pathogenic variant in three subjects and as a mono-allelic variant in two subjects.

Among the 22 probands from group I segregating milder hearing loss, four subjects (18.2%) carried the p.V37I variant of *GJB2*, either as one of two mutant alleles (three subjects) or as a single heterozygote (one subject). In contrast, only one (1.2%) of the 81 probands from group II with a severe hearing loss carried the p.V37I variant (as a single heterozygote) (Table 1). The prevalence of p.V37I allele in group I was significantly higher than that in group II (p = 0.008, Fisher’s exact test). It was also higher than that of the published Korean normal hearing control group (p = 0.001, Fisher’s exact test) (Table 1). However, the prevalence of the p.V37I allele in group II was not significantly different to that of the published Korean normal hearing control (p = 0.731, Fisher’s exact test).

**Table 1:** Differences in prevalence of p.V37I variant and inheritance patterns.

		Group I*	Group II**	Control group†
**AR or sporadic (family)**		22	81	3418
**p.V37I carrier (n)**		4	1	39
**Allele frequency**		0.091	0.006	0.005
**Onset of hearing loss (mean value)**		6.33 yr	<1 yr	−
**Fisher’s exact test (p-value)**	vs Group II	0.008	−	−
	vs Control	0.001	0.731	−

* Group I: slight, mild or moderate degree of hearing loss;

** Group II: Severe or profound degree of hearing loss;

† Control group: adapted from three independent studies with the same ethnic background (Han et al., 2008; Kim et al., 2011, Park et al, 2000).

**Figure 2:**
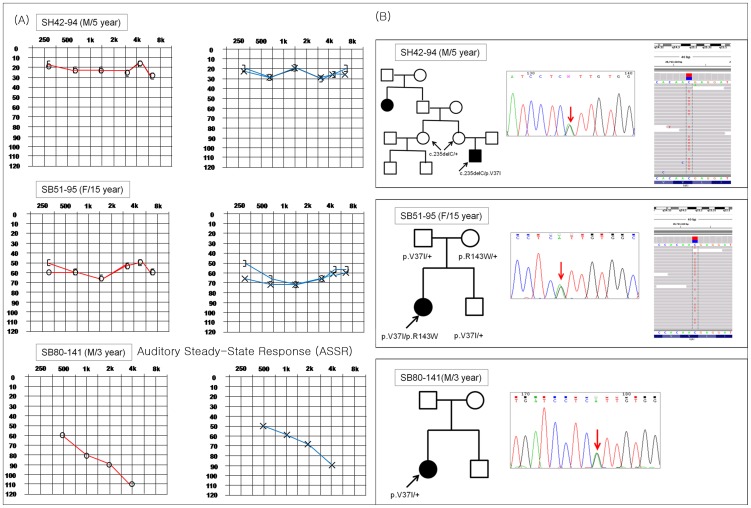
Three families with the p.V37I variant and moderate or slight hearing loss. Two subjects (SH42-94, SB51-95) carried the p.V37I allele *in a trans* configuration with another mutant allele of *GJB2,* as a compound heterozygote and the other subject (SB80-141) carried the p.V37I allele as the only variant. (A) Detailed data of each subject’s pure tone audiometry at their initial visit. (B) Detailed data of each subject’s pedigree and Sanger sequencing traces. Next generation sequencing trace of p.V37I variant of *GJB2* from two subjects (SH42-94, SB51-95) are shown. The Auditory Steady-State Response (ASSR) result of SB80-141 at the initial visit is shown due to poor cooperation in pure tone audiometry test.

**Figure 3:**
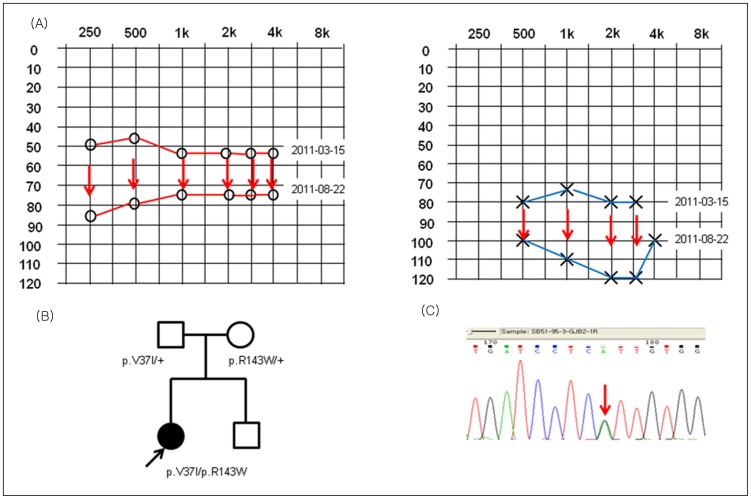
Progressive hearing loss shown in a family. (A) Pure tone audiometry (at initial visit, and at follow up visit 5 months later) (B) Subjects’ pedigree and (C) Sanger sequencing traces are shown. Rapid progression of hearing loss over 5 months was noted in SHJ12 carrying the p.V37I and p.R143W mutant alleles.

Breaking down the four subjects from group I (SH42-94, SB51-95, SB80-141 and SHJ12) who carried the p.V37I variant, three subjects (SH42-94, SB51-95, SHJ12) carried the p.V37I allele in a *trans* configuration with another mutant allele of *GJB2* in the other homologous chromosome, as a compound heterozygote. SB80-141 carried the p.V37I allele as the only variant (Figure 2B). The p.R143W allele was in a *trans* configuration with the p.V37I in the other homologous chromosome for two compound heterozygotes (SB51-95 and SHJ12), while c.235delC was in a *trans* configuration with p.V37I in one subject (SH42-94) with subtle hearing loss. Rapid progression of hearing loss over a 5 month period was noted from SHJ12 carrying the p.V37I and p.R143W mutant alleles (Figure 3). Hearing loss of these four subjects was first detected at an average of 6.33 years old (Table 1).

**Figure 4:**
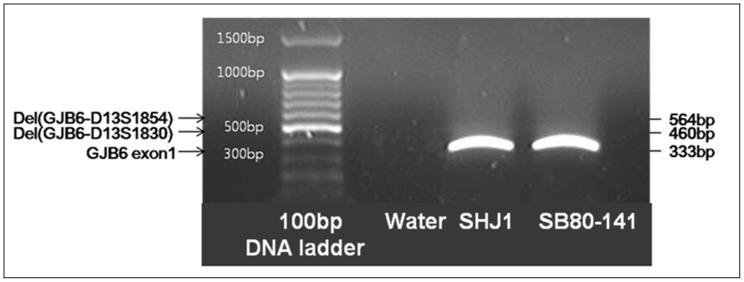
Multiplex breakpoint PCR to detect del(GJB6-D13S1830) and del(GJB6-D13S1854) ) from two single heterozygous carriers of p.V37I (SHJ1 and SB80-141). A 564 bp band and a 460 bp band, indicative of the amplification of the del (GJB6-D13S1854) breakpoint junction and del (GJB6-D13S1830) breakpoint junction, respectively, were absent in SHJ1 and SB 80–141, despite a positive band being amplified from the control region, GJB6 exon 1.

We further verified that the hearing loss could be accounted for by the p.V37I allele of *GJB2*, and not by other known deafness genes by targeted next generation sequencing (panel sequencing) of 82 known deafness genes (including *GJB2, GJB3* and *GJB6*). For two subjects (SH42-94 and SB51-95), the other 81 known deafness genes were excluded as causes of the observed hearing loss (Figure 2B, Table 2, and Table S1). Two and three novel SNPs were detected from SH42-94 and SB51-95, respectively (Table 2). However, no potentially pathogenic candidate variants compatible with recessive inheritance of hearing loss were found from the two subjects (Table 2). Unfortunately, we were not able to perform targeted next generation sequencing of the 82 deafness genes for SB80-14 carrying the p.V37I as the only variant since the parents of SB80-14 were reluctant for the next generation sequencing at that moment. Instead, the possibility of the presence of at least two reported large deletions (del(GJB6-D13S1830) and del(GJB6-D13S1854)) in a *trans* configuration with the p.V37I from SB80-141 and SHJ1 was excluded; no band compatible with the presence of the two large deletions, despite a clear amplification from the exon 1 of GJB6, was observed (Figure 4).

**Table 2:** Variants detected by targeted next generation sequencing of 82 deafness genes from two subjects (SH42-94, SB51-95) carrying p.V37I variant of GJB2.

Patient	freq(38)	Type	Gene	Annotation	Chr	Start	End	Ref	Var	Genotype	Quality score	1000 g	dbsnp135
**SH42-94**	2	Nonsyn	DSPP	NM_014208:exon5:c.C3429A:p.D1143E	4	88537243	88537243	C	A	Hetero	10.26	−	−
	2	fs. Del	GJB2	NM_004004:exon2:c.235delC:p.L79fs	13	20763486	20763486	G	−	Hetero	99	0.0023	rs80338943
	2	Nonsyn	GJB2	NM_004004:exon2:c.G109A:p.V37I	13	20763612	20763612	C	T	Hetero	99	0.01	rs72474224
	2	Nonsyn	TRIOBP	NM_001039141:exon7:c.C1494G:p.D498E	22	38120057	38120057	C	G	Hetero	71.06	−	−
**SB51-95**	2	Nonsyn	DSPP	NM_014208:exon5:c.C3429A:p.D1143E	4	88537243	88537243	C	A	Hetero	99	−	−
	1	Nonsyn	TMC1	NM_138691:exon10:c.C505G:p.P169A	9	75357411	75357411	C	G	Hetero	99	−	−
	2	Nonsyn	GJB2	NM_004004:exon2:c.C427T:p.R143W	13	20763294	20763294	G	A	Hetero	99	0.0005	rs80338948
	2	Nonsyn	GJB2	NM_004004:exon2:c.G109A:p.V37I	13	20763612	20763612	C	T	Hetero	99	0.01	rs72474224
	2	Nonsyn	TRIOBP	NM_001039141:exon7:c.C1494G:p.D498E	22	38120057	38120057	C	G	Hetero	77.16	−	−

One subject (SHJ1) carrying a single heterozygous p.V37I allele from group II was a monozygotic twin. The monozygotic twin brother of SHJ1 carried the same p.V37I allele but had completely normal hearing, indicating that detection of the p.V37I allele in this subject with profound deafness is probably incidental (Table 1).

## Discussion

To the best of our knowledge, the frequency of the p.V37I allele has never been reported in an exclusive group with only mild hearing loss. The carrier frequency of the p.V37I allele was calculated as 18.2% among Korean subjects with moderate or slight hearing loss in this study. This figure is strikingly high, considering that the carrier frequency of the p.V37I variant from Korean normal hearing control populations ranged from 0.88 to 1.25% [12], [13]. This figure is also comparable to what was reported in a recent study by Li et al. (2012) in a Chinese Han population. They showed that p.V37I exclusive genotype of *GJB2* was present in 20% of postnatal permanent childhood hearing impairment subjects [23]. However, they did not specify the degree of hearing loss for the postnatal permanent childhood hearing impairment subjects. Delayed onset hearing loss is likely to manifest as mild or moderate hearing loss rather than severe or profound hearing loss, because postnatal permanent childhood hearing impairment subjects were defined when they passed the newborn hearing screening or the pediatric hearing tests, but developed bilateral hearing loss before the age of 9 years. This implies that the frequency of p.V37I carrier would be around 20%, overall, in Southeast Asian populations with mild to moderate postlingual hearing loss. Our study cohort excluded families segregating autosomal dominant hearing loss and subjects older than 15 years of age, leaving only pediatric hearing impaired subjects, mostly without any family history of hearing loss, in our final cohort. The high carrier rate, up to 18.2%, of the p.V37I variant from this selected cohort may justify genetic testing focusing upon the p.V37I variant of GJB2, when the pediatric subjects show moderate or slight hearing impairment without any sign of syndromic hearing impairments or any inner ear anomaly, and if they show sporadic or autosomal recessive hereditary patterns.

The significantly higher prevalence of the p.V37I variant of *GJB2* in the mild hearing loss group compared with the severe hearing loss group and the reported Korean normal hearing control group, provides strong genetic evidence of its contribution to the pathogenesis of mild hearing loss, rather than more severe or profound hearing loss. This agrees with previous descriptive reports where p.V37I homozygotes and c.235delc/p.V37I compound heterozygotes had mild to moderate nonsyndromic hearing loss [19], [26] [27].

The significantly variable degree of hearing loss among the p.V37I carriers in our cohort (Figure 2A) led us to hypothesize that the phenotype detected in some p.V37I carriers in the literature might be caused by other autosomal deafness genes, and that the detection of p.V37I in these patients was incidental. Our families were not large enough to perform linkage analysis to identify a candidate locus. In this study, we tried to minimize such a possibility by target exome sequencing of 82 deafness genes from two carriers of p.V37I (SH42-94 and SB51-95) with substantially different degrees of hearing loss between them (see Figure 2A). After completing our filtering strategy, two or three coding region variants remained, except *GJB2* variants, but none of the variants were compatible with a recessive inheritance pattern. More specifically, p.D1143E in DSPP and p.D498E in TRIOBP (Table 2) were identified as polymorphisms in the Korean population. However, it is still possible that a totally different gene not included in the 82 deafness genes or a non-coding region mutation in the 82 deafness genes may be responsible for the milder phenotype of some p.V37I carriers, especially when p.V37I is detected as the single variant.

Two single heterozygotes (SB80-141 from group I and SHJ1 from group II) of p.V37I were detected in our cohort. We suggest a different interpretation of the genotype depending upon the degree of hearing loss. Based upon our data, p.V37I detected as a single heterozygote is most likely to contribute to the phenotype if hearing loss is mild or moderate. It is not surprising that a substantial portion of DFNB1 patients carry only one detectable mutation of *GJB2* [25], [28]. There could be other mutations within the DFNB1 locus, outside of the *GJB2* coding regions, such as a large deletion involving *GJB6* [25], [29]. We used breakpoint PCR to detect the reported large genomic deletions in the DFNB1 locus, but none were detected (Figure 4). Unreported novel deletions within this locus might reside in a *trans* configuration with p.V37I in SB80-141 from group I. In contrast, the detection of this variant in congenital profound hearing loss, such as in SHJ1 in our study, is most likely to be fortuitous and a search for other etiologies is necessary. First, a monozygotic twin brother of SHJ1, who also carries the p.V37I allele, has perfectly normal hearing, strongly suggesting this allele has nothing to do with the observed hearing loss in SHJ1. Non-penetrance of the p.V37I allele could also be possible. However, our interpretation favors the fortuitous detection of this allele in congenital profound deafness, which agrees with our data that the carrier frequency of the variant (1.2%) in group II was not significantly different from that of the reported Korean normal hearing control group.

Occasionally, the p.V37I variant can be expressed as progressive hearing loss, especially in combination with other pathogenic mutations of *GJB2* in a *trans* configuration. Our subject SHJ12, carrying p.V37I and p.R143W, showed a rapid progression of hearing loss over 5 months (Figure.3), eventually requiring cochlear implantation. Such patients tend to show delayed onset of hearing loss, which cannot be detected by the newborn hearing screening test. Therefore, delayed diagnosis and delayed hearing rehabilitations can result. Thus, regular follow up is required in moderate or slight hearing loss with the p.V37I variant of *GJB2*. Some researchers advocate newborn genetic screening (NGS) [12], [30] to avoid missing subjects with delayed on-set hearing loss related to this mutant allele. However, the carrier rate of this allele in normal controls is as high as 1.2% in the Korean population, making it difficult to interpret the result (especially when p.V37I is detected as only a single heterozygous allele) without information on the exact hearing status, not just an information of ‘pass’ or ‘refer’ obtained by the new born hearing screening. More practically, we suggest checking the p.V37I variant of *GJB2* in children with mild to moderate hearing impairment that segregates in a manner compatible with autosomal recessive inheritance. Further study is needed to elucidate the function of the p.V37I mutation, not only the p.V37I phenotype, but also any genetic interaction with other pathogenic or causative variants.

## Conclusions

Our study strongly suggests that the p.V37I variant of *GJB2* contributes to the pathogenesis of milder hearing loss and may justify sequencing of *GJB2* from subjects with mild to moderate degree of hearing loss in the Korean population.

## Supporting Information

Table S1 **Statistics for targeted next generation sequencing from the two subjects with p.V37I variant.**
(DOCX)
