# Supplementary material for: Prevalence of p.V37I Variant of GJB2 in Mild or Moderate Hearing Loss in a Pediatric Population and the Interpretation of Its Pathogenicity
Source: PLoS One. 2013 Apr 25;8(4):e61592. doi: 10.1371/journal.pone.0061592 (PMC3636207; doi:10.1371/journal.pone.0061592)
Supplement: Table S1 — Statistics for targeted next generation sequencing from the two subjects with p.V37I variant. (DOCX) [file pone.0061592.s001.docx]

**Table S1.** Statistics for targeted next generation sequencing from the two subjects with p.V37I variant

| **Sample** | **Depth** | | | | | | | **SNP** | | | | **Indel** | **Novel SNP** | | | | **Novel Indel** |
| --- | --- | --- | --- | --- | --- | --- | --- | --- | --- | --- | --- | --- | --- | --- | --- | --- | --- |
|  | **Mean** | **Q3** | **Median** | **Q1** | **%>=10** | **%>=50** | **%>=100** | **Total** | **synon** | **nonsyn** | **stopgain** | **Total** | **Total** | **synon** | **nonsyn** | **stopgain** | **Total** |
| SH42-94 | 583.22 | 500 | 500 | 280 | 95.2 | 94.1 | 92.1 | 223 | 134 | 89 | 0 | 8 | 12 | 9 | 3 | 0 | 1 |
| SB51-95 | 628.47 | 500 | 500 | 334 | 95.2 | 94 | 92.2 | 209 | 130 | 79 | 0 | 9 | 19 | 14 | 5 | 0 | 0 |
| **Average** | 605.8 | 500.0 | 500.0 | 307.0 | 95.2 | 94.1 | 92.2 | 216.0 | 132.0 | 84.0 | 0.0 | 8.5 | 15.5 | 11.5 | 4.0 | 0.0 | 0.5 |
| **s.d.** | 32.0 | 0.0 | 0.0 | 38.2 | 0.0 | 0.1 | 0.1 | 9.9 | 2.8 | 7.1 | 0.0 | 0.7 | 4.9 | 3.5 | 1.4 | 0.0 | 0.7 |
